# Occlusion de la veine centrale de la rétine du sujet jeune révélant une hyperhomocystéinémie: à propos d'un cas

**DOI:** 10.11604/pamj.2015.21.296.7674

**Published:** 2015-08-24

**Authors:** Wafa Ammari, Olfa Berriche

**Affiliations:** 1 Service d'Ophtalmologie, Hôpital Tahar Sfar, Mahdia, Tunisie; 2 Service de Médecine Interne, Hôpital Tahar Sfar, Mahdia, Tunisie

**Keywords:** Occlusion, veine, homcysteine, occlusion, vein, homcysteine

## Image en medicine

L'occlusion de la veine centrale de la rétine (OVCR) du sujet jeune est une entité clinique différente de celle du sujet âgé. Elle est moins associée aux facteurs de risque cardiovasculaires et l'étiologie spécifique reste souvent inconnue. L'hyperhomocystéinémie est connue pour être un facteur de risque rare Nous rapportons le cas d'un patient âgé de 25 ans ayant une baisse brutale et indolore de l'acuité visuelle de l'oeil gauche. L'examen ophtalmologique a retrouvé une acuité visuelle à 10/10^e^, P2 à droite et à compte les doigts à 1m à gauche non améliorable. L'examen des segments antérieurs était normal aux deux yeux. Le fond d'oeil gauche retrouvait un oedème papillo-rétinien; des veines dilatées et tortueuses; et des hémorragies superficielles en flammèche et profondes en nappe. L'angiographie rétinienne à la fluorescéine confirma le diagnostic d'une (OVCR) gauche, forme mixte à prédominance oedémateuse. La tomographie en cohérence optique (OCT) montre un oedème maculaire cystoïde (OMC) responsable de l'acuité visuelle effondrée. Un bilan étiologique demandé (bilan lipidique complet, bilan inflammatoire, bilan immunologique, bilan de thrombophilie) était normal. Par ailleurs, le taux plasmatique de l'homocystéine était élevé à 19,2 μmol/L. Un traitement à base de vitaminothérapie a été commencé. L'évolution était marquée par une amélioration de l'acuité visuelle à 2/10, P8 avec régression partielle de l'OMC.

**Figure 1:**
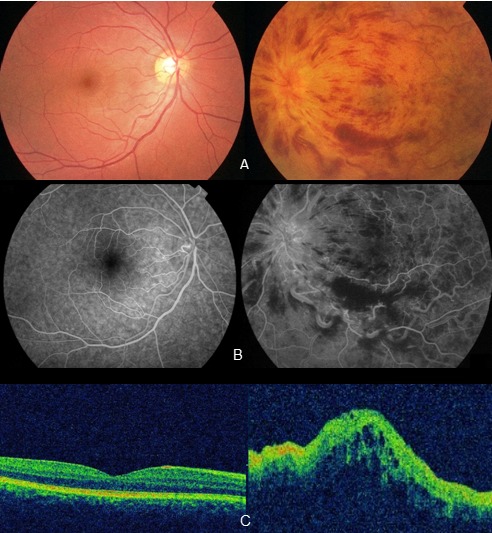
(A) le fond d’œil (FO) gauche: oeœdème papillo-rétinien; des veines dilatées et tortueuses et des hémorragies superficielles en flammèche et profondes en nappe; (B) l'angiographie rétinienne à la fluorescéine: Occlusion de la veine centrale de la rétine (OVCR) gauche, forme mixte à prédominance œdémateuse; (C) La tomographie en cohérence optique (OCT): un œdème maculaire cystoïde (OMC) avec une épaisseur maculaire centrale (EMC) = 408,1µm

